# Protective role of agomelatine via modulation of TLR4/NF-κB: NLRP3/IL-1β signaling pathways in testosterone-induced benign prostatic hyperplasia in rats

**DOI:** 10.1007/s00210-025-04874-6

**Published:** 2025-12-19

**Authors:** Asmaa Mohamed Abdel-Aziz, Sara M. Ahmed, Walaa Yehia Abdelzaher, Nada Amgad Mohamed, Alyaa E. Abdelkader, Rasha Fouad Ahmed, Asmaa Mohamed Mahmoud Ali, Al Shimaa Mahmoud Kotb, Alyaa Abdelfattah Abdelmonaem

**Affiliations:** 1 Department of Medical Pharmacology, Faculty of Medicine, Minia University, Minya, 61519 Egypt; 2 Department of Histology and Cell Biology, Faculty of Medicine, Minia University, Minya, 61519 Egypt; 3 Department of Medical Microbiology and Immunology, Faculty of Medicine, Minia University, Minya, 61519 Egypt; 4 Department of Biochemistry, Faculty of Medicine, Minia University, Minya, 61519 Egypt; 5 Department of Forensic Medicine & Clinical Toxicology, Faculty of Medicine, Minia University, Minya, 61519 Egypt; 6 Department of Physiology, Faculty of Medicine, Minia University, Minya, 61519 Egypt; 7 Faculty of Nursing, Lotus University, Minya, 61768 Egypt

**Keywords:** Agomelatine, Anti-inflammatory, Anti-oxidative, Benign prostatic hyperplasia, Rats

## Abstract

Benign prostatic hyperplasia (BPH) is a prevalent urological disorder in aging men, frequently linked to hormonal fluctuations. Agomelatine (AGO), a melatonergic receptor agonist and serotonin 2C antagonist, has demonstrated anti-inflammatory and antioxidant properties. This study aimed to explore the potential of AGO in mitigating BPH induced by testosterone propionate (TP) in rats. Male rats received TP (5 mg/kg/day, subcutaneously) to induce BPH and were pretreated with AGO (80 mg/kg/day) for 28 days. AGO inhibited the rise in prostate index, serum prostate-specific antigen (PSA), and testosterone levels compared to the BPH-only group. Histological analysis revealed that AGO significantly improved the pathological alterations in prostate tissue architecture when compared to BPH-only group. Furthermore, AGO notably reduced oxidative stress induced by TP, evidenced by a decrease in lipid peroxidation and preserved levels of reduced glutathione. This reduction in oxidative stress led to a decrease in pro-inflammatory cytokines in the prostate, such as Toll-like receptor 4 (TLR4) and interleukin-1 beta (IL-1β). Additionally, AGO caused a marked decrease in of NLRP3 inflammasome and vascular endothelial growth factor-A (VEGF-A) protein expressions. When compared to the BPH group, AGO’s effects also included a reduction in the immunoexpression of nuclear factor kappa B (NF-κB) and myeloid differentiation primary response 88 (MyD88) proteins. The findings from this study provide new evidence that AGO can alleviate testosterone-induced BPH in rats, likely through the inhibition of the TLR4/NFκB and NLRP3/IL-1β signaling pathways. These results suggest that AGO could be a promising therapeutic option for managing BPH.

**Graphical Abstract:**

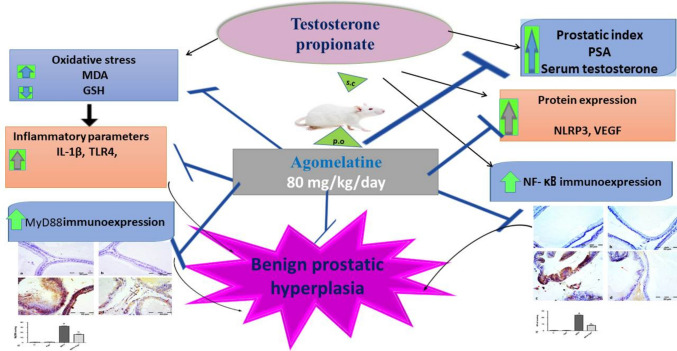

## Introduction

Benign prostatic hyperplasia (BPH) is a prevalent condition among men, with its incidence rising as age increases. Approximately 8% of men aged 40 are affected, with prevalence reaching around 50% in those aged 60 and older. BPH is characterized by the increased growth of prostatic epithelial and stromal cells (Cannarella et al. 2021).

The exact molecular mechanisms that contribute to the development of BPH remain unclear. A variety of risk factors have been linked to this condition, including oxidative stress, inflammation, aging, and androgen stimulation. Growing evidence indicates that androgens and inflammation interact in intricate ways that influence prostatic tissues (Abdel-Aziz et al. 2020).

According to Tong et al. (2020), androgens may have a significant impact on inflammation, which could subsequently alter androgen activity. Multiple studies have shown that oxidative products and a depletion of antioxidants play roles in BPH. Inflammation of the prostate can lead to the generation of free radicals and oxidative stress, potentially resulting in cellular proliferation and contributing to BPH development (Minciullo et al. 2015).

The NOD-like receptor family pyrin domain-containing 3 (NLRP3) inflammasome has been associated with various diseases, particularly chronic inflammatory disorders (He et al. 2020). A clinical study found elevated levels of inflammasome components in prostate tissues from BPH patients, suggesting that inflammasomes play a crucial role in mediating prostate inflammation associated with this condition (Kashyap et al. 2015).

When cellular stress is detected, NLRP3 activation recruits caspase-1-dependent inflammatory cytokines, such as Toll-like receptor 4 (TLR4), interleukin-1β (IL-1β), IL-18, tumor necrosis factor-α (TNF-α), and activated nuclear factor-κB (NF-κB). In human BPH specimens with inflammatory infiltrates, TLR4 mRNA and protein expression are significantly elevated compared to non-inflamed BPH tissues, and these elevated TLR4 levels correlate with increased inflammatory markers. These inflammatory responses can create a feedback loop of inflammation and cellular proliferation within the prostate, further contributing to its enlargement (Liu et al. 2018).

Research indicates that inappropriate activation of the NLRP3 inflammasome may drive cellular proliferation and tumor development; however, its specific role in BPH remains to be fully elucidated (Yu et al. 2020).

Several studies have underscored the significance of the enzyme 5-alpha reductase in BPH pathogenesis, as it converts testosterone to dihydrotestosterone in target tissues. As a result, 5-alpha reductase inhibitors, such as finasteride and dutasteride, are considered standard treatments for BPH (Zhu and Imperato-McGinley 2009).

Despite their effectiveness, these medications are associated with a variety of side effects, including gynecomastia, upper respiratory infections, sexual dysfunction, decreased libido, and changes in sperm DNA, motility, and count (Patel and Chapple 2008; Irwig and Kolukula 2011; Chiba et al. 2011). Some patients may find these side effects unacceptable, leading them to decline finasteride as a treatment option for their BPH. Consequently, there is a pressing need for new and equally effective therapies, especially for patients who are hesitant to use finasteride for managing their condition.

Agomelatine (AGO) is an atypical antidepressant that functions as an agonist at melatonin (MT1 and MT2) receptors and as an antagonist at serotonin 2C (5-HT2C) receptors. While primarily studied for its antidepressant effects, emerging evidence suggests that it may also have significant antioxidant and anti-inflammatory effects by inhibiting the NLRP3 inflammasome and the release of inflammatory cytokines. This action could help regulate the chronic inflammatory processes linked to BPH pathogenesis, particularly in patients who may also suffer from mood disorders (Khalaf et al. 2020; Rossetti et al. 2022).

Previous data had reported the presence of MT receptors in the rat prostate. Via AGO activation of MT1 receptor with consequent attenuation of calcium influx induced by sex steroids and its anti-gonadotrophic effect by inhibiting testosterone synthesis in the testis, it could ameliorate ROS production and increase the antioxidant defense mechanism, with attenuation of cytokine release creating an anti-proliferative environment, preventing disease progression (Gobbo et al. 2015; Fotovat et al. 2022; Megerian et al. 2023).

In light of previous findings regarding the efficacy of AGO in reducing oxidative stress and inflammation in tissues, the current study was designed to explore its potential protective effects in testosterone-induced BPH in adult male albino rats. This investigation will focus on the mechanisms underlying its action, specifically examining the TLR4/NF-κB; NLRP3/IL-1β signaling pathways.

## Materials and methods

### Chemicals

AGO was obtained in powder form from the Inspire Pharmaceutical Co., Egypt. Testosterone propionate (TP) was sourced from Cid Company, Egypt. ELISA kits for rat testosterone, IL-1β, TLR-4, and prostate-specific antigen (PSA) were procured from Boster Biological Technology (USA, testosterone kit) and Elabscience Biotechnology Inc. (USA, for the other kits). The primary antibodies used were rabbit anti-NF-κB polyclonal antibody (1:1000, bs-0465R, Bioss Company, USA) and rabbit anti-MyD88 polyclonal antibody (1:200, DF6162, Affinity Biosciences, USA). The primary antibodies for NLRP3 and vascular endothelial growth factor-A (VEGF-A) were rabbit anti-NLRP3 (1:1000, ab263899, Abcam, UK) and rabbit anti-VEGF-A (1:1000, ab46154, Abcam, UK).

### Animals

The study was conducted using 24 adult male Wistar albino rats, each weighing 200–250 g and aged 8–10 weeks, obtained from the National Research Center (Giza, Egypt). The rats were housed in groups of three per cage, with ad libitum access to pelleted feed and drinking water (El-Nasr Co., Cairo, Egypt). They were allowed to acclimatize to the laboratory conditions for 2 weeks before the experiment began. The environment was maintained at 24 ± 2 °C with a 12-h light/dark cycle. Animal procedures followed the ARRIVE guidelines and the NIH Guide for the Care and Use of Laboratory Animals. The study was approved by the Faculty of Medicine, Minia University, Egypt (approval number 677: 3/2023).

### Experimental design

Twenty-four male albino rats were randomly allocated into four groups (six rats per group): group 1 (control group): received 1 ml/kg of saline, the vehicle for AGO (p.o.), and 1 ml/kg of olive oil, the vehicle for TP (s.c.);

group 2 (AGO group): received AGO (80 mg/kg/day, p.o.) for 28 days (Khalaf et al. 2020);

group 3 (BPH group): was administered TP (5 mg/kg/day, s.c.) for 28 days to induce BPH (Abdel-Aziz et al. 2020); and.

group 4 (BPH + AGO group): received TP (5 mg/kg/day, s.c.) for 28 days and AGO (80 mg/kg/day, p.o.) for 28 days concurrently.

### Sample collection and storage

At the conclusion of the experiment, animals were weighed and anesthetized with ketamine (50 mg/kg, intramuscularly) (Ibrahim et al. 2021). Blood was drawn from the jugular vein and centrifuged (Jantezki centrifuge, T30, Germany) at 5000 rpm for 10 min to separate serum, which was stored at − 20 °C for further analysis. The prostate of each rat was quickly dissected, cleaned with saline, and weighed. Each prostate was divided into three sections: one was fixed in formalin for histopathological and immunohistochemical analysis, the second was frozen for Western blot analysis, and the third was homogenized in phosphate-buffered solution (10% homogenate) and stored at − 80 °C for biochemical analysis.

### Estimation of prostate index

The prostate from each rat was quickly removed and weighed, and the prostate index was calculated as the ratio of prostate weight to body weight (mg/g) (Vasilyeva et al. 2019).

### Biochemical analysis

#### Assessment of prostatic oxidative stress parameters

Reduced glutathione (GSH) levels in the prostate were measured by a method involving the reduction of Elman’s reagent (Shaker 2014). The resulting yellow color was assessed spectrophotometrically at 412 nm. GSH levels were quantified using a standard curve and expressed as µmol/g tissue.

Malondialdehyde (MDA) levels in prostate tissue were determined by the thiobarbituric acid method, as described by Buege and Aust (1978). This method measures thiobarbituric acid-reactive substances, which correlate with MDA concentration. Absorbance was recorded at 535 nm using a Beckman DU-64 spectrophotometer (USA). MDA levels were calculated from a standard curve and expressed as nmol/g tissue.

#### Assessment of pro-inflammatory parameters

Prostatic IL-1β and TLR4 levels were quantified using commercial ELISA kits according to the manufacturer’s protocols (Catalog Nos: E-EL-R0012 and E-EL-R0990, respectively).

#### Assessment of serum testosterone and PSA levels

Serum testosterone and PSA levels were quantified using commercial ELISA kits according to the manufacturer’s protocols (Catalog Nos: EK7014 and E-EL-R0796, respectively).

### Western blotting analysis

Prostate tissue homogenates (30 μg total protein) were boiled in a loading buffer containing 2-mercaptoethanol and loaded onto a 12% SDS-PAGE gel, which was run for 2 h at 100 V. Proteins were then transferred to PVDF membranes and blocked with 5% non-fat milk and 0.05% Tween-20 in TBS-T for 1 h. Membranes were incubated overnight at 4 °C with primary antibodies: anti-NLRP3 (1:1000 dilution, Abcam, UK), anti-VEGF-A (1:1000 dilution, Abcam, UK), and β-actin (Santa Cruz Biotechnology, USA). The secondary antibody, goat anti-rabbit polyclonal conjugated to horseradish peroxidase (1:5000 dilution, Cell Signaling Technology, USA), was applied. Protein bands were visualized by chemiluminescence and quantified densitometrically using ImageJ software (Li et al. 2021).

### Histopathological and immunohistochemistry assessment

#### Histological examination

Following sacrifice, the ventral lobes of the prostate from all experimental groups were fixed in 10% neutral-buffered formalin for 24 h, followed by storage in 70% ethanol. Tissues were dehydrated in graded alcohols, cleared in xylene, embedded in paraffin, and sectioned into 5–6-µm-thick slices. These sections were stained with hematoxylin and eosin for histological evaluation. The histopathological scoring and assessment is performed via the semi-quantitative analysis described by (Shackelford et al. 2002). The evaluation is divided into five grades: score 1, reflects minimal hyperplasia (< 1%); score 2, reflects slight hyperplasia (1–25%); score 3, reflects moderate hyperplasia (25–50%); score 4, reflects moderately severe hyperplasia (51–75%); and score 5, reflects severe hyperplasia (75–100%).

#### Immunohistochemical analysis for NF-κB and MyD88

Paraffin sections were dewaxed, rehydrated, and washed with phosphate-buffered saline (PBS). Endogenous peroxidase activity was blocked with H₂O₂, and antigen retrieval was achieved by boiling sections in citrate buffer. The sections were incubated overnight at 4 °C with primary antibodies: anti-NF-κB (1:1000 dilution, Bioss Company, USA) and anti-MyD88 (1:200 dilution, Affinity Biosciences, USA). After incubation, the sections were washed, treated with secondary antibody (goat anti-rabbit IgG), and stained using the streptavidin–biotin complex. The chromogenic substrate 3,3′-diaminobenzidine (DAB) was used to develop the color, and the sections were counterstained with hematoxylin (Cemek et al. 2008).

##### Photography

The sections were photographed and analyzed for positive expression of NF-κB and MyD88. Positive expression was indicated by brown staining in the cytoplasm and/or nucleus for NF-κB and cytoplasmic staining for MyD88. Negative controls were included by substituting PBS for the primary antibody. Immunohistochemical staining was quantified using ImageJ 22 software.

##### Morphometric analysis

For the morphometric assessment, the mean number of NF-κB positive nuclei was manually counted, and Image J software (version 1.25-win-java8) was used to measure the mean area fraction of MyD88-positive cells in immunohistochemically stained sections X400 in randomized non overlapping 10 fields.

### Statistical analysis

Data were analyzed using one-way ANOVA followed by Tukey’s multiple comparison test. Normal distribution of the quantitative variables was tested first by Shapiro–Wilk test and *p* value was more than 0.05 reflecting a normally distributed data. Ordinal data were analyzed by the use of the Kruskal–Wallis test followed by Dunn’s multiple comparison test. The results are presented as means ± SEM. Statistical analysis was performed using GraphPad Prism software (version 5), with *p*-values less than 0.05 considered statistically significant.

## Results

### Effect of AGO on prostatic index in testosterone-induced BPH in rats

Administration of TP caused a significant increase in prostatic index compared to control and AGO groups. Co-administration of AGO with TP significantly decreased prostatic index compared to BPH group (Table 1).

**Table 1:** Effect of AGO on prostatic index and prostatic oxidative stress parameters in testosterone-induced BPH in rats

Groups	Prostatic index	Prostatic GSH (µmol/g tissue)	Prostatic MDA (nmol/g tissue)
C	**0.22 ± 0.008**	**40.94 ± 1.59**	**5.25 ± 0.25**
AGO	**0.21 ± 0.007**	**43.88 ± 1.45**	**5.98 ± 0.22**
BPH	**0.35 ± 0.012**^**ab**^	**13.31 ± 1.59**^**ab**^	**16.7 ± 0.55**^**ab**^
BPH + AGO	**0.26 ± 0.019**^**c**^	**33.58 ± 0.99**^**c**^	**8.31 ± 0.31**^**c**^

Values represent the mean ± SEM (*n* = 5–6). Results are considered significantly different when *p* < 0.05

^a^Significantly different from control group

^b^Significantly different from AGO group

^c^Significantly different from BPH group [*C* control group, *AGO* agomelatine, *BPH* benign prostatic hyperplasia group, *GSH* reduced glutathione, *MDA* malondialdehyde]

### Effect of AGO on prostatic oxidative stress parameters in testosterone-induced BPH in rats

BPH group showed a significant decrease in prostatic level of GSH with a significant increase in prostatic MDA level when compared to control and AGO groups. On the other hand, BPH + AGO group significantly increased prostatic GSH level and decreased prostatic MDA level when compared to BPH group (Table 1).

### Effect of AGO on prostatic pro-inflammatory parameters in testosterone-induced BPH in rats

In Table 2, BPH group showed significant increase in prostatic IL-1 β and TLR4 levels when compared to control and AGO groups. Meanwhile, BPH + AGO group significantly improved prostatic anti-inflammatory parameters in comparison to BPH group.

**Table 2:** Effect of AGO on serum level of testosterone, PSA and prostatic pro-inflammatory parameters in testosterone-induced BPH in rats

Groups	Testosterone (ng/mL)	IL-1β (pg/mL)	TLR4 (ng/mL)	PSA (ng/mL)
C	1.07 ± 0.05	32.51 ± 0.49	0.53 ± 0.02	0.54 ± 0.02
AGO	1.02 ± 0.05	31.57 ± 0.36	0.58 ± 0.01	0.56 ± 0.02
BPH	3.47 ± 0.18^ab^	52.47 ± 0.93^ab^	0.94 ± 0.02^ab^	0.89 ± 0.03^ab^
BPH + AGO	2.47 ± 0.12^c^	46.16 ± 0.70^c^	0.79 ± 0.01^c^	0.74 ± 0.02^c^

Values represent the mean ± SEM (*n* = 5–6). Results are considered significantly different when *p* < 0.05

^a^Significantly different from control group

^b^Significantly different from AGO group

^c^Significantly different from BPH group [*C* control group, *AGO* agomelatine, *BPH* benign prostatic hyperplasia group, *IL-1β* interleukin-1β, *TLR4* Toll-like receptor 4, *PSA* prostatic specific antigen]

### Effect of AGO on serum testosterone and PSA levels in testosterone-induced BPH in rats

Administration of TP caused a significant increase in serum testosterone level and PSA levels compared to control and AGO groups. Co-administration of AGO with TP significantly decreased serum testosterone and PSA levels when compared to BPH group (Table 2).

### Effect of AGO on prostatic NLRP3 and VEGF—an expression in testosterone-induced BPH in rats

**Fig. 1:**
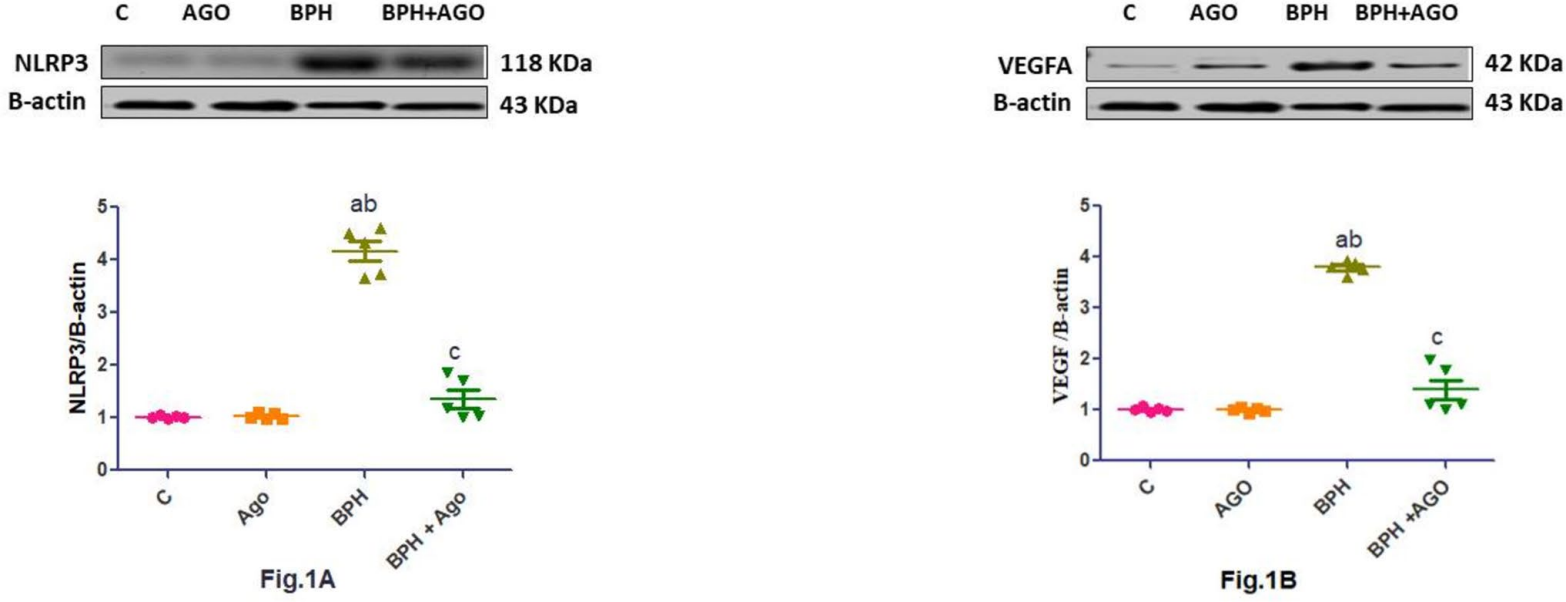
Effect of AGO on prostatic NLRP3 and VEGF expression in testosterone-induced BPH in rats. Values represent the mean ± SEM (*n* = 5–6). Results are considered significantly different when *p* < 0.05. (**a**) Significantly different from control group, (**b**) significantly different from AGO group, and. (**c**) significantly different from BPH group [C, control group; AGO, agomelatine; BPH, benign prostatic hyperplasia group; NLRP3, the NOD-like receptor family pyrin domain-containing 3 inflammasome; VEGF-A, vascular endothelial growth factor-A]

Administration of TP caused a significant increase in protein expression of prostatic NLRP3 and VEGF-A compared to control and AGO groups. Co-administration of AGO with TP significantly decreased the expression of NLRP3 and VEGF-A compared to BPH group (Fig. 1A, B).

### Effect of AGO on the prostatic histopathological pattern in testosterone-induced BPH in rats

#### H&E results

**Fig. 2:**
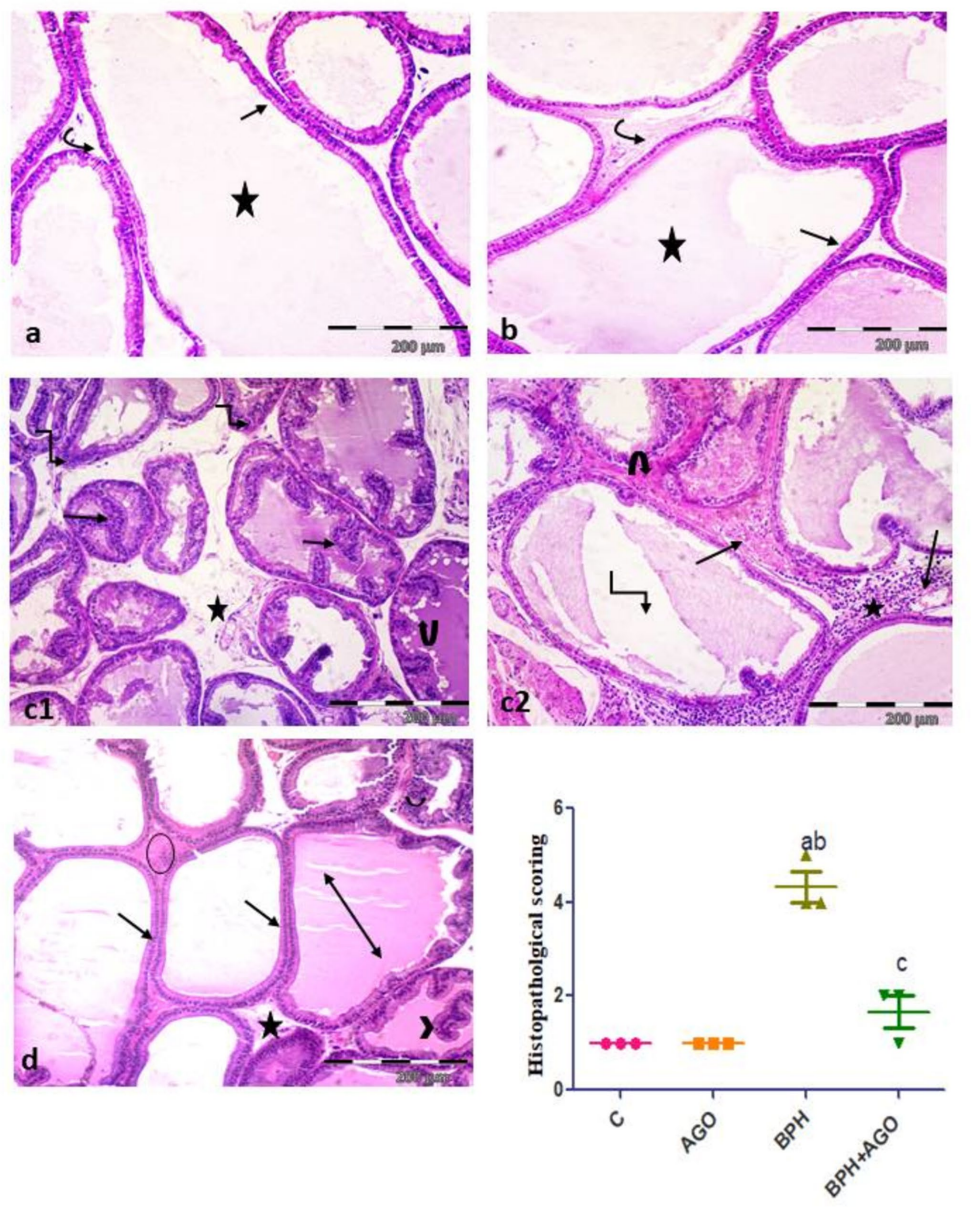
Photomicrographs of prostatic sections stained with hematoxylin, and eosin. Representative photomicrographs of prostatic sections stained with H& E. **a**, **b** Control group and AGO group, respectively, showing closely packed prostatic acini lined by simple columnar epithelium (arrows), filled with homogenous acidophilic secretions (asterisk), and are separated by thin fibromuscular stroma (curved arrow). BPH group (**c1**), showing irregular shaped acini separated by wide stromal spaces (asterisk), many long papillary folds narrowing their lumina (arrows), stratification of acinar lining epithelium (elbow arrows), and vacuolated acinar secretions (curved arrow). BPH group (**c2**), showing acini surrounded with stromal acidophilic material (curved arrow), congested blood vessels with marginal inflammatory cells (arrows), and intense inflammatory cellular infiltration (asterisk), notice shrinkage of prostatic secretions (elbow arrow). **d** BPH + AGO group showing most prostatic acini are regular and lined with simple columnar epithelium (arrows) and some with stratified epithelium (curved arrow) or forming papillary projections (arrow head); acini are filled with homogenous acidophilic secretions (double headed arrow), and separated with thin fibromuscular stroma (asterisk), few inflammatory cellular infiltration are seen in stroma (circle). (H&EX100, scale bar = 200 ϻm). **e** Semiquantitative analysis of H&E prostatic hyperplasia 5 grades scoring system. Results are considered significantly different when *p* < 0.05. (**a**) Significantly different from control group, (**b**) significantly different from AGO group, and (**c**) significantly different from BPH group. C, control group; AGO, agomelatine; BPH, benign prostatic hyperplasia

Sections of control and AGO groups showed closely packed prostatic acini lined with simple columnar epithelium, filled with homogenous acidophilic secretions. Acini were separated by thin fibromuscular stroma (Fig. 2a, b). BPH group revealed irregular prostatic acini with numerous long branched papillary folds narrowing their lumina and many acini showed stratification of their epithelial lining. Acini were separated by very wide stroma. Thick acidophilic material was demonstrated surrounding some acini. Congested blood vessels with marginal inflammatory cells and intense inflammatory cellular infiltration were observed within wide stroma. Some acini showed either vacuolated secretions or shrinkage of their secretions (Fig. 2c1, c2). BPH + AGO group showed improvement in the histological appearance of most prostatic acini in the form of reduced epithelial height (lined with simple columnar epithelium), diminished papillary folds, and lumina filled with homogenous acidophilic secretions. Some acini still showed papillary folds and stratified epithelial lining. Few inflammatory cells were observed within thin fibromuscular stroma (Fig. 2d).

The semiquantitative analysis according to the 5 grades scoring system revealed that the prostate hyperplasia in the BPH group was significantly increased compared to control group (all *P* < 0.001). Meanwhile, BPH + AGO group significantly improved compared to the BPH group (all *P* < 0.001) (Fig. 2e).

#### NF-κB immunohistochemical results

**Fig. 3:**
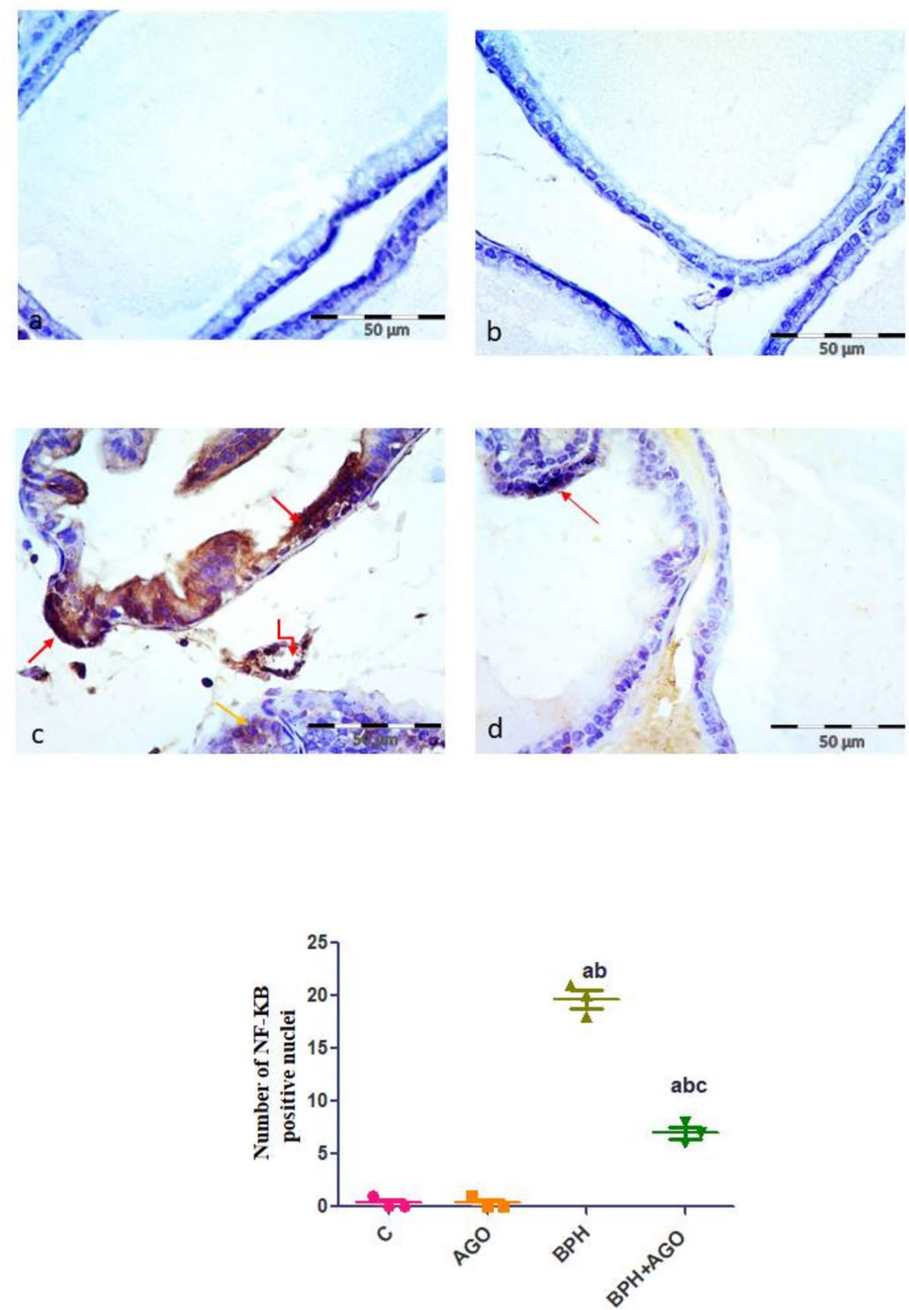
Evaluation of immunohistochemical expression of prostatic NF-Κb. Representative photomicrographs of NF-κβ immuno-stained sections of rat prostate from **a** and **b**, control and AGO groups, respectively, showing negative immunoreaction of most epithelial cells lining the acini (red arrows), faint cytoplasmic immunoreaction in few epithelial cells (black arrows). **c** BPH group, showing strong positive nuclear immunoreaction of epithelial cells lining the acini (red arrows), positive cytoplasmic immune reaction in few epithelial cells lining the acini (yellow arrow), and endothelial cells lining blood vessels in stroma (elbow arrow). **d** BPH + AGO group, showing positive nuclear immune reaction in few epithelial cells lining the acini (red arrow). Immunostaining for NF-κβ, Original magnification (× 400). **e** Quantitative analysis of the mean number of NF-κβ positive nuclei, Results are considered significantly different when *p* < 0.05. (**a**) Significantly different from control group, (**b**) significantly different from AGO group, and (**c**) significantly different from BPH group. C, control group; AGO, agomelatine; BPH, benign prostatic hyperplasia

Sections of control and AGO groups showed negative immunoreaction of epithelial cells lining the acini (Fig. 3a, b), while BPH group showed dense nuclear expression in the most of acinar epithelial cells, cytoplasmic expression in few epithelial cells, and endothelial cells lining blood vessels in stroma (Fig. 3c). BPH + AGO group showed positive nuclear immune reaction in few epithelial cells lining the acini (Fig. 3d).

#### MyD88 immunohistochemical results

**Fig. 4:**
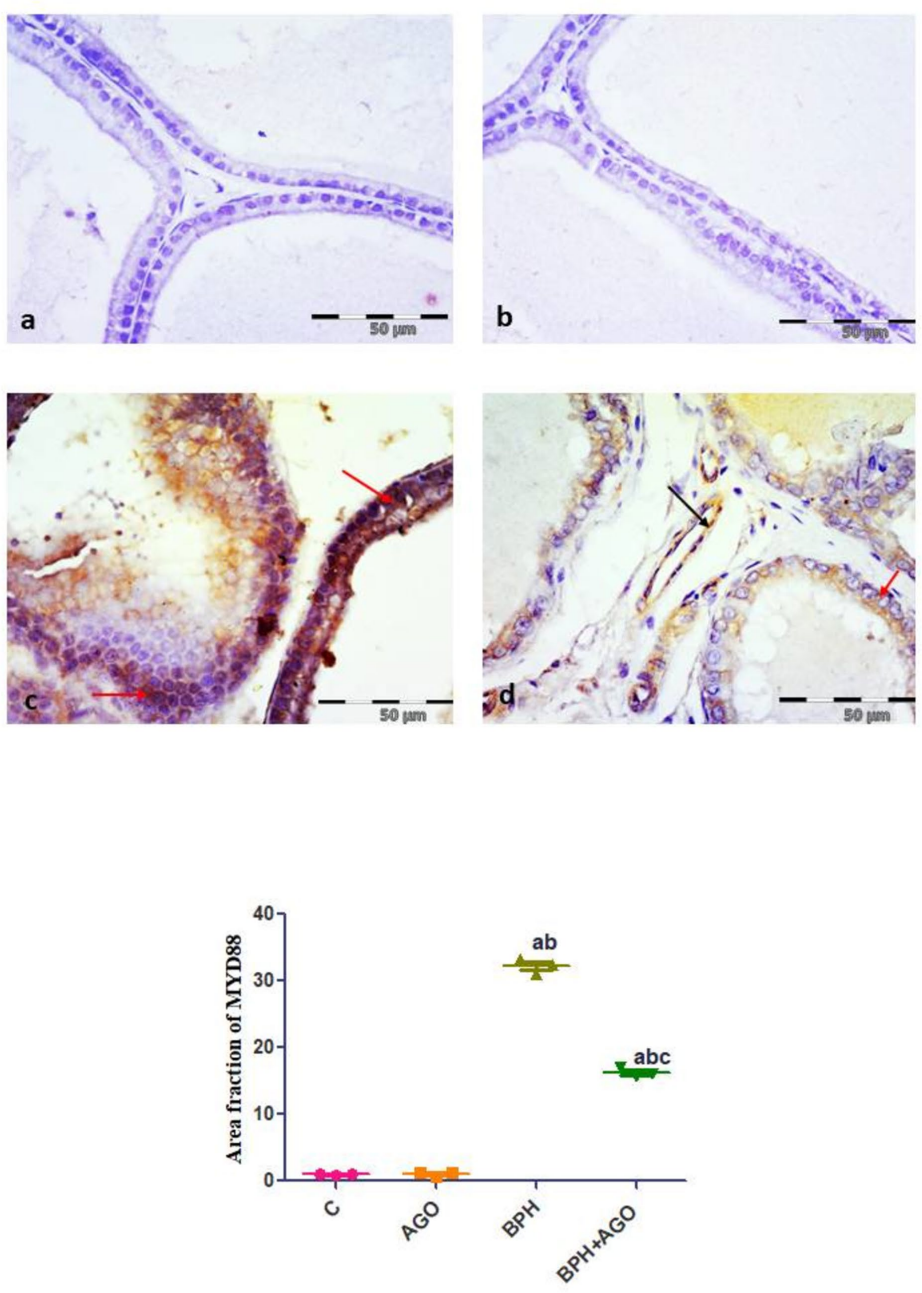
Evaluation of immunohistochemical expression of prostatic MyD88. Representative photomicrographs of MyD88 immuno-stained sections of rat prostate from **a** and **b**, control and AGO groups, respectively, showing negative immunoreaction of epithelial cells lining the acini. **c** BPH group, showing strong positive cytoplasmic immunoreaction of epithelial cells lining the acini (red arrows). **d** BPH + AGO group, showing mild positive cytoplasmic immune reaction in scattered acinar cells (red arrow) and endothelial cells lining blood vessels in stroma (black arrow). Immunostaining for MyD88, original magnification (× 400). **e** Quantitative analysis of the mean area fraction of MyD88 positive cells. Results are considered significantly different when *p* < 0.05. (**a**) Significantly different from control group, (**b**) significantly different from AGO group, and (**c**) significantly different from BPH group. C, control group; AGO, agomelatine; BPH, benign prostatic hyperplasia

Prostatic sections from control and AGO groups showed negative immunoreaction of epithelial cells lining the acini (Fig. 4a, b). BPH group showed dense cytoplasmic immunoreaction of most of epithelial cells lining the acini (Fig. 4c). BPH + AGO group showed mild positive cytoplasmic immune reaction in scattered acinar cells and endothelial cells lining blood vessels in stroma (Fig. 4d).

Morphometric results of the mean number of NF-κB nuclear positive cells and the mean area fraction of MyD88-positive cells showed significant increase in the BPH group compared to control group (all *P* < 0.001). Meanwhile, there was a significant decrease in the BPH + AGO group compared to the BPH group (all *P* < 0.001) (Figs. 3e and 4e).

## Discussion

BPH is a prevalent condition in older men, with its incidence rising progressively with age, leading to a decreased quality of life. Severe complications associated with BPH include acute urinary retention, urinary tract infections, bladder stones, and even renal failure (Li et al. 2018). The pathogenesis of BPH involves factors such as oxidative stress, inflammation, and an imbalance between cell proliferation and apoptosis (Eid and Abdel-Naim 2020).

Over the past decade, AGO has attracted considerable attention due to its anti-inflammatory effects, which are believed to stem from its ability to scavenge free radicals, enhance antioxidant enzyme activity, and suppress both pro-oxidant enzymes and inflammatory signaling pathways (Khalaf et al. 2020). This study aimed to evaluate AGO’s potential in preventing testosterone-induced BPH in rats and to investigate the underlying mechanisms.

In the current study, AGO exhibits a potent protective activity in BPH rats manifested by inhibition of the rise in prostate index, serum PSA, and testosterone levels. Furthermore, AGO notably reduced oxidative stress, evidenced by a decrease in lipid peroxidation and preserved levels of reduced glutathione. This reduction in oxidative stress led to a decrease in pro-inflammatory cytokines in the prostate, such as TLR4 and IL-1β. Additionally, AGO caused a marked decrease in NLRP3 inflammasome and VEGF-A protein expressions. When compared to the BPH group, AGO’s effects also included a reduction in the immunoexpression of NF-κB and MyD88 proteins causing a significant reduction in prostatic cell hyperplasia.

The treatment with AGO for 28 days effectively counteracted the testosterone-induced increase in prostate index. Moreover, AGO reduced the effects of prostatic hyperplasia, as shown by a significant decline in serum levels of prostate-specific antigen (PSA) and testosterone. These results are consistent with earlier studies, which have demonstrated that elevated PSA levels are associated with a higher incidence of BPH and can be used as a reliable marker for prostatic volume in monitoring the disease (Abdel-Aziz et al. 2020). PSA testing is somewhat predictive of prostate volume. Benign prostates of 35 cc size will typically generate a PSA of 1.5 ng/mL (Ng et al. 2024). Testosterone is known to be a key player in BPH development, facilitating the transformation of normal prostate tissue into hyperplastic tissue, which could eventually progress to prostate cancer (Bartsch et al. 2000). Moreover, testosterone treatment increased the risk of receiving a diagnosis of BPH in hypogonadal malesozda (Fendereski et al. 2025). AGO’s ability to normalize both serum PSA and testosterone levels in rats with BPH suggests its potential in preventing BPH progression and its associated biomarkers.

Moreover, improvements in histopathological findings further reinforce the protective role of AGO. The prostate tissues from AGO-treated rats exhibited considerable improvements in their histological structure, showing significant reductions in prostatic hypertrophy and hyperplasia, as well as in the formation of intraluminal papillary projections within the glandular acinar epithelium. This runs hand in hand with Yu et al. (2018) that mentioned that injecting melatonin into Syrian hamster testes during the breeding period significantly decreases the content of testosterone, decreases testicular volume, and diminishes androgen synthesis. The protective effect of AGO may be attributed to its activation of melatonin receptors (Khalaf et al. 2020). Melatonin is recognized for its potent antioxidant properties and its role in regulating lipid metabolism. It has also been shown to be crucial in inhibiting tumor growth, particularly in prostate cancer, highlighting its anticancer potential (Cipolla-Neto et al. 2014).

Tai et al. (2016) found that reduced melatonin levels in the morning are strongly linked to the development of prostate cancer. Similarly, Wang et al. (2012) demonstrated that melatonin could reduce the metastasis of prostate cancer. Zhou et al. (2021) also emphasized that melatonin positively affects the long-term survival of prostate cancer patients with poor prognosis. These findings suggest that AGO, as an agonist of melatonin receptors, may serve as a promising target for further research, particularly in the context of BPH and prostate cancer.

Numerous studies have established the role of oxidative stress in the development of testosterone-induced BPH (Hata et al. 2023). Testosterone increases oxidative stress by reducing SOD, glutathione peroxidase, and catalase activities, which increase lipoperoxidation in the testis (Nolasco-Pérez et al. 2025). Chronic inflammation and oxidative stress were consistently identified within hyperplastic prostate tissue. Excessive ROS production, diminished antioxidant defense, and sustained cytokine release create a proproliferative and antiapoptotic environment, accelerating disease progression (Kaltsas et al. 2025). In this study, AGO exhibited significant antioxidant effects by reducing the prostatic tissue levels of MDA and significantly increasing the levels of GSH. These results align with previous research that has demonstrated the protective effects of various antioxidants in mitigating BPH progression (Wang et al. 2021; Aljehani et al. 2022; Jin et al. 2023). Furthermore, AGO’s antioxidant role in a variety of disease conditions has been widely recognized in several studies (Rebai et al. 2021; Cherngwelling et al. 2021; Ozdamar et al. 2022; Alruhaimi et al. 2023).

Since 1937, many studies have sought to clarify the connection between chronic inflammation and BPH, proposing that BPH could be an immune-mediated inflammatory condition. Changes in the complex network of cytokines and growth factors are believed to play a crucial role in the inflammatory processes within the prostate. This ongoing tissue damage and repair can lead to sustained stimulation of stromal and epithelial cells, which may contribute to BPH development (De Nunzio et al. 2016). Localized inflammation is often associated with BPH, at least histologically. While the exact etiology is unclear, possible causes include increased detrusor voiding pressure, obesity, low-grade or chronic prostatitis, compression of the prostatic ducts, and autoimmune disorders. Studies have confirmed that NSAIDs can improve BPH symptoms (Kahokehr et al. 2013; Ng et al. 2024).

Previous studies have also suggested that both prostatic epithelial and stromal cells express receptors for cytokines, with epithelial cells notably expressing various Toll-like receptors (TLRs), including TLR-2, TLR-4, TLR-5, and TLR-9. These TLRs are key regulators of inflammation, and upon activation, they recruit downstream signaling molecules such as MyD88. This, in turn, leads to the activation of NF-κB and the upregulation of the NLRP3 inflammasome, processes that have been linked to the release of pro-inflammatory cytokines like IL-1β, which contribute to fibrosis and tissue remodeling in BPH (Nickel et al. 2008; Abdelzaher et al. 2021).

In the present study, testosterone treatment significantly elevated the levels of TLR4 and IL-1β in prostatic tissue, along with increased immunoexpression of NF-κB and MyD88. Additionally, there was a notable rise in the protein levels of NLRP3 in the prostate. These findings align with previous studies (De Nunzio et al. 2016; Zhang et al. 2021; Jin et al. 2023). Also a recent study revealed that testosterone induced elevation in inflammatory markers IL-6, IL-1β, TNF-α, TGF-β, and NF-κB and oxidative stress (increase in MDA and decrease in GSH) and elevation of Rho kinase1 (Aljorani et al. 2025).

However, pretreatment with AGO resulted in a significant reduction in these inflammatory markers. Specifically, AGO decreased the prostatic tissue levels of TLR4 and IL-1β, as well as reduced the immunoexpression of NF-κB and MyD88. AGO also notably lowered the prostatic protein levels of NLRP3, which is likely attributed to its anti-inflammatory properties. These results are consistent with Chumboatong et al. (2022), who observed that AGO exerted its anti-inflammatory effects by inhibiting the TLR4/NLRP3 pathway in rats. Several other studies have also highlighted AGO’s anti-inflammatory effects in various pathological conditions (Dobrek et al. 2015; Eraslan et al. 2020; Savran et al. 2020; Ozmen et al. 2022; Alruhaimi et al. 2023).

Furthermore, AGO demonstrated a substantial effect on prostate pathophysiology by reducing the expression of VEGF-A, a key mediator of angiogenesis in BPH. By lowering VEGF-A expression, AGO may inhibit the hyperplastic growth of the prostate (Abdel-Aziz et al. 2020). Gautam et al. (2018) reported that VEGF expression level might have an important role in the prediction of prostate cancer as it was significantly differed with BPH. In addition, VEGF expression level showed intense staining in the tissue samples with higher grading of prostate cancer which reveals its importance as prognostic marker.

In the current study, the BPH group showed a marked increase in VEGF-A expression compared to the control group. However, pretreatment with AGO significantly decreased VEGF-A expression relative to the BPH group, suggesting that AGO can limit excessive vascularization and stromal cell proliferation in the prostate, thereby alleviating BPH-related tissue alterations. These results are consistent with previous research indicating AGO’s ability to suppress VEGF-A expression (Cheng et al. 2020; Yuksel et al. 2023; Lin et al. 2023).

**Fig. 5:**
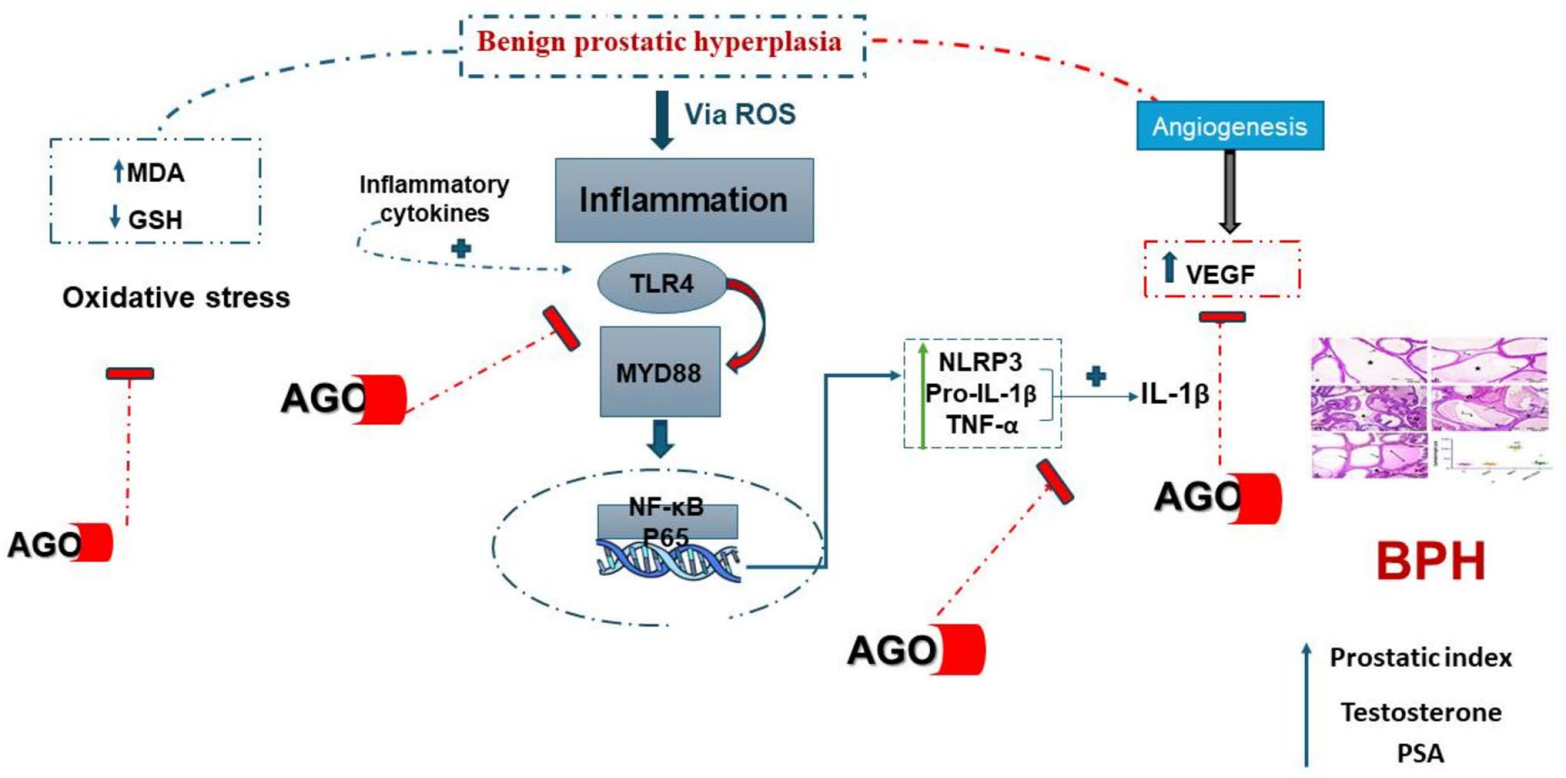
Schematic diagram showing the proposed molecular mechanisms explaining the protective effects of agomelatine (AGO) in testosterone-induced BPH in rats. GSH, reduced glutathione; MDA, malondialdehyde; NLRP3, the NOD-like receptor family pyrin domain-containing 3 inflammasome; VEGF-A, vascular endothelial growth factor-A; IL-1β, interleukin-1β; TLR4, Toll-like receptor 4; PSA, prostatic specific antigen; NF-κB, nuclear factor kappa; and MyD88, myeloid differentiation primary response 88

In summary, AGO exhibits potent antioxidant activity and modulates multiple pro-inflammatory pathways, including TLR4, MyD88, NF-κB, IL-1β, and NLRP3. Additionally, AGO directly suppresses VEGF-A, contributing to a reduction in prostatic cell hyperplasia. These combined effects suggest that AGO could be a promising therapeutic agent for managing BPH (Fig. 5). The limitations of our study were the lack of facility to investigate melatonin receptor expression in the prostate. However, the current study revealed a valuable protective role for a specific melatonin receptor agonist against testosterone-induced BPH in rats which may offer a new indication for AGO therapy.

## Conclusion

This study highlights the multifaceted protective effects of AGO in the management of BPH. By mitigating oxidative stress, inflammation, and hormonal imbalances, AGO contributes to restoring a healthier prostatic environment and slowing the progression of BPH. The results underscore AGO’s potential as a novel therapeutic agent for BPH, acting through the modulation of key signaling pathways such as TLR4/MyD88/NF-κB and the NLRP3/IL-1β inflammasome. Additionally, AGO’s impact on angiogenesis, particularly via the suppression of VEGF-A, further supports its therapeutic promise. Overall, this study provides compelling evidence for AGO’s potential as a promising treatment option for patients suffering from BPH.

## Data Availability

All source data for this work (or generated in this study) are available upon reasonable request.
